# Recurrent hemorrhagic pancreatic pseudocyst with superior mesenteric vein thrombosis treated by pancreaticoduodenectomy and vein graft reconstruction: a case report

**DOI:** 10.1097/RC9.0000000000000160

**Published:** 2026-02-05

**Authors:** Takashi Miyata, Taigo Nagayama, Hisashi Nishiki, Koichi Okamoto, Hideto Fujita, Hiroyuki Takamura

**Affiliations:** Department of General and Digestive Surgery, Kanazawa Medical University Hospital, Ishikawa, Japan

**Keywords:** case report, hemorrhage, interventional radiology, pancreaticoduodenectomy, pancreatic pseudocyst, superior mesenteric vein thrombosis

## Abstract

**Introduction and importance:**

Hemorrhage into a pancreatic pseudocyst is rare but potentially fatal. Interventional radiology-guided transcatheter arterial embolization (TAE) is often first-line therapy; however, rebleeding is common, and surgery may be required. Inflammation from pancreatitis can make surgery technically challenging, underscoring the importance of careful management.

**Case presentation:**

A 68‑year‑old Japanese man with chronic pancreatitis and heavy alcohol use presented with acute abdominal pain. Computed tomography revealed a 25 mm hemorrhagic cystic lesion in the pancreatic head containing a pseudoaneurysm, superior mesenteric vein (SMV) thrombosis, and inflammatory fat stranding. Initial TAE controlled bleeding from a branch of the dorsal pancreatic artery, but recurrent hemorrhage occurred 7 months later, requiring repeat TAE. Three months later, another episode from the anterior superior pancreaticoduodenal artery was embolized. Because of repeated bleeding and persistent SMV thrombosis, pancreaticoduodenectomy with SMV resection and reconstruction was performed using an external iliac vein graft under an Anthron‑coated catheter shunt. The postoperative course was uneventful, and the patient has remained symptom‑free for 2 years.

**Clinical discussion:**

This case illustrates the complexity of managing hemorrhagic pancreatic pseudocyst with SMV thrombosis and recurrent bleeding. While endovascular therapy can provide temporary control, repeated episodes may necessitate definitive surgery. An intraoperative Anthron-coated catheter shunt enabled safe SMV reconstruction in this case despite extensive thrombosis.

**Conclusion:**

Surgical intervention should be considered in select patients with recurrent hemorrhage and complicating vascular pathology. This case adds to the limited literature and may inform future treatment strategies.

## Introduction

Hemorrhage into a pancreatic pseudocyst is an uncommon yet life-threatening complication, with a reported mortality rate of 25%–45%^[^1,2^]^. Prompt diagnosis and appropriate management are essential to improve outcomes. Interventional radiology (IVR)-guided arterial embolization is often the first-line treatment^[^3,4^]^; however, rebleeding rates remain high, and many patients ultimately require surgery[5]. Dense adhesions and severe inflammation that develop after pancreatitis make surgery technically demanding, and the risk of postoperative pancreatic fistula must be considered.

**HIGHLIGHTS**

- Hemorrhage into a pancreatic pseudocyst is a rare but critical condition.
- Transcatheter arterial embolization is performed first, but rebleeding is common.
- Our patient experienced repeat rebleeding after multiple embolizations.
- An Anthron-coated catheter shunt was created.
- Pancreaticoduodenectomy with superior mesenteric vein reconstruction was successful.

We report a rare case of recurrent hemorrhage into a pancreatic pseudocyst, in which hemostasis was attempted three times via IVR. Definitive treatment was achieved by pancreaticoduodenectomy (PD) with superior mesenteric vein (SMV) reconstruction using an external iliac vein graft, with a pre-established Anthron-coated (Toray Co., Ltd., Tokyo, Japan) catheter shunt. This case highlights the surgical challenges posed by SMV thrombosis and repeated bleeding and adds to the limited literature on optimal management strategies for this rare but critical condition.

## Case presentation

A 68-year-old Japanese man with chronic pancreatitis and hyperuricemia presented with sudden-onset severe abdominal pain. He had a history of heavy alcohol consumption and long-standing abdominal discomfort. On arrival, he was afebrile (36.6°C), hypertensive (165/83 mmHg) and had upper abdominal guarding. Laboratory tests revealed leukocytosis (15 600/μL), elevated C-reactive protein (4.3 mg/dL), and hyperamylasemia (500 U/L). Tumor marker concentrations (i.e., carcinoembryonic antigen and carbohydrate antigen 19–9) were within normal limits.

**Figure 1.**
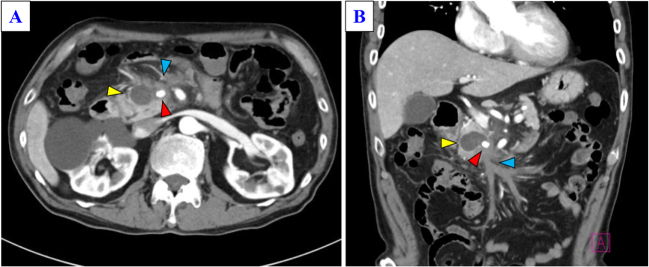
Contrast-enhanced computed tomography (CT) findings. (A) Axial and (B) coronal contrast-enhanced abdominal CT images at the initial presentation showing a 2.5 cm pseudocyst in the pancreatic head (yellow arrowhead) with a hyperintense area suggestive of intracystic hemorrhage (red arrowhead). The superior mesenteric vein (SMV) was extensively thrombosed (blue arrowhead).

**Figure 2.**
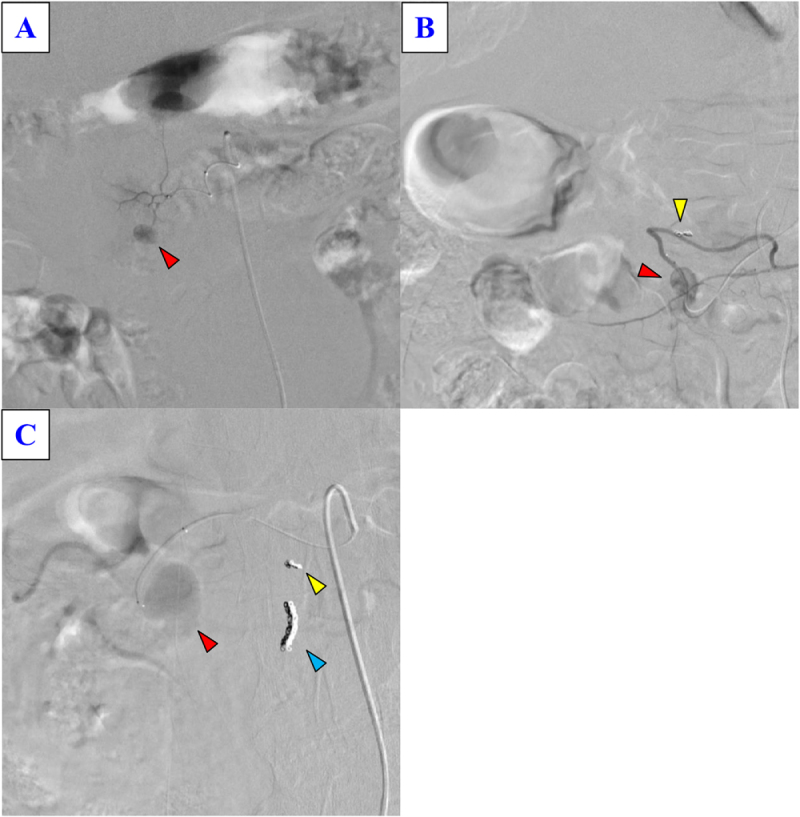
Selective angiography. (A) During the first episode, contrast extravasation from the dorsal pancreatic artery was observed (red arrowhead). (B) During the second episode, recurrent extravasation from the dorsal pancreatic artery was noted (red arrowhead); the yellow arrowhead indicates the initial coiling. (C) During the third episode, extravasation from the anterior superior pancreaticoduodenal artery was detected (red arrowhead). The yellow arrowhead indicates the first coiling, and the blue arrowhead indicates the second coiling.

Contrast-enhanced computed tomography showed a 25 mm hemorrhagic cystic lesion in the pancreatic head, likely arising from a branch of the dorsal pancreatic artery, and a 5 cm segment of thrombosed SMV with surrounding inflammatory fat stranding (Fig. 1A and B, Supplemental Digital Content Figure S1, available at: http://links.lww.com/IJSCR/A5). At presentation, differential diagnoses such as malignancy were considered less likely, and the findings were attributed to pancreatitis-related venous thrombosis with arterial bleeding. Emergency transcatheter arterial embolization (TAE) was performed for the culprit artery (Fig. 2A), and the patient was discharged after 2 weeks. Following discharge, alcohol abstinence therapy was initiated as part of conservative management.

Seven months later, he presented with similar symptoms, and repeat TAE was performed for the same arterial branch. Three months later, another bleeding episode occurred, this time from a branch of the anterior superior pancreaticoduodenal artery, which was also embolized (Fig. 2B and C). Considering the repeated hemorrhages, recurrent SMV thrombosis, and the patient’s preference, surgical management was planned.

**Figure 3.**
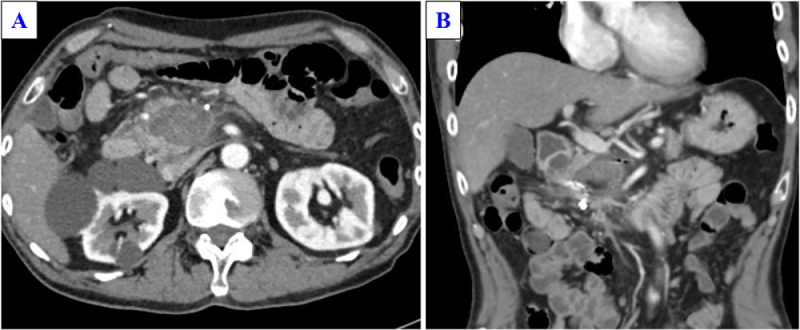
Preoperative computed tomography (CT) findings. (A) Axial and (B) coronal abdominal CT images immediately before surgery showing a 4.0 cm mass in the pancreatic head with features suggestive of thrombosis. Extensive occlusion of the superior mesenteric vein (SMV) was persistent, and the vein was not visualized.

Preoperative computed tomography revealed a 40 mm mass-like lesion in the pancreatic head with internal thrombosis and persistent SMV occlusion (Fig. 3A and B). Although portal vein flow may have been maintained by collateral circulation, PD with SMV resection and reconstruction using an external iliac vein graft, with an Anthron-coated catheter shunt, was scheduled.

**Figure 4.**
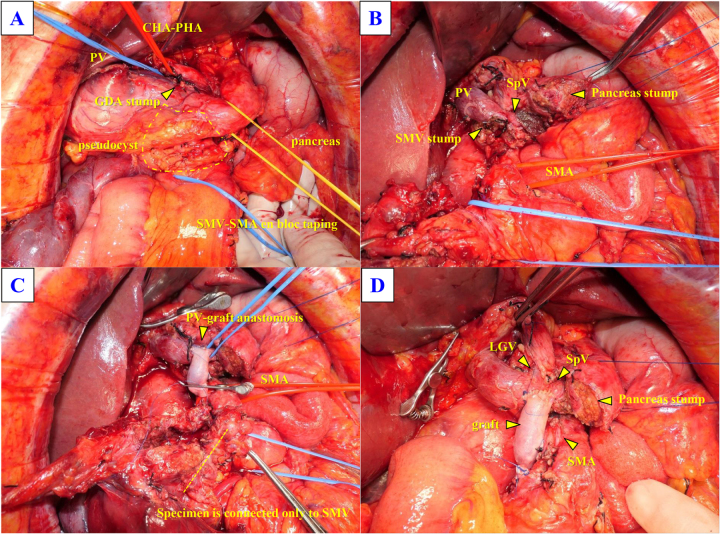
Intraoperative findings. (A) The pseudocyst was located in the pancreatic head. Because of severe inflammation, the superior mesenteric vein (SMV) and superior mesenteric artery (SMA) could not be easily dissected and were temporarily encircled with tapes. At this stage, the gastroduodenal artery (GDA), stomach, and small intestine had already been divided. (B) The pancreas was transected above the portal vein (PV), and the confluence of the SMV and PV was exposed. The specimen was dissected from the SMA while preserving the perivascular nerve plexus. (C) During dissection, the cyst wall was inadvertently punctured. The specimen was completely separated from the SMA, remaining attached only to the SMV. PV graft reconstruction was completed at this point, followed by SMV division and specimen removal. (D) Operative field after pseudocyst resection and SMV reconstruction. CHA, common hepatic artery; PHA, proper hepatic artery; SpV, splenic vein; LGV, left gastric vein.

Intraoperatively, a shunt was established using an Anthron-coated catheter between the ileal vein and the right great saphenous vein. Following gastric transection, hepatoduodenal ligament dissection, and small bowel transection, the mesocolon was divided. The SMV and superior mesenteric artery (SMA) were taped en bloc. The pancreas was transected above the portal vein, and the SMV–portal vein confluence was divided. The SMA was dissected free from the specimen, with preservation of the perivascular nerve plexus. The cyst wall was opened during dissection, but the specimen was completely mobilized from the SMA. SMV reconstruction with the vein graft was performed before specimen removal (Fig. 4A–D).

The operative time was 735 minutes, with blood loss of 2070 mL. Intraoperative transfusion comprised 4 units of red blood cells, 4 units of fresh frozen plasma, and 10 units of platelets.

**Figure 5.**
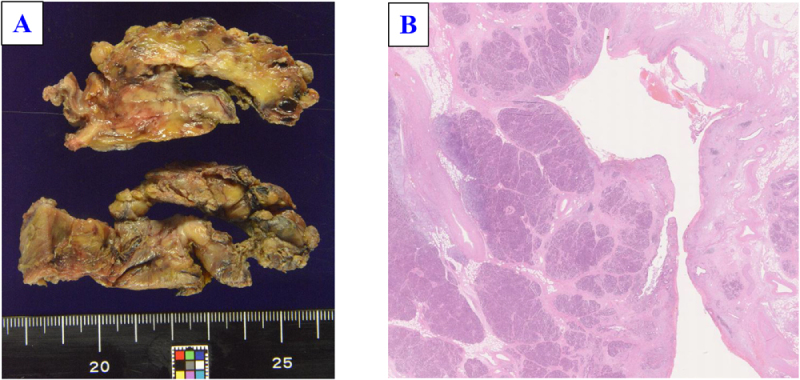
Gross and histopathological findings. (A) Gross examination of the resected specimen revealed a punctured cystic lesion in the pancreatic head, with brownish exudate adherent to the inner surface. (B) Histopathological examination showed that the cyst wall consisted of fibrous to fibrogranulomatous tissue with inflammatory cell infiltration. Partial communication with the pancreatic duct was observed, but most of the cyst wall lacked epithelial lining, consistent with a pseudocyst. No malignant features were identified. Hematoxylin and eosin staining; 30× magnification.

Gross examination revealed a 40 mm cystic lesion in the pancreatic head (Fig. 5). Histology confirmed a pseudocyst with partial epithelial lining and communication with the pancreatic duct, without evidence of malignancy or cyst–portal vein communication. The postoperative course was uneventful, and the patient was discharged on postoperative day 21. Postoperative follow-up consisted of continued alcohol abstinence and periodic imaging surveillance. At the time of writing, he has remained symptom free for 2 years (see Supplemental Digital Content Table S1, available at: http://links.lww.com/IJSCR/A6).

## Discussion

Pancreatic pseudocysts develop in approximately 10%–15% of patients following acute or chronic pancreatitis and are encapsulated by inflammatory exudate and fibrous tissue^[^6,7^]^. While some pseudocysts resolve spontaneously, intracystic hemorrhage is rare (6%–10%) and associated with the highest mortality rate among pseudocyst-related complications^[^1,2^]^.

The mechanisms of bleeding in pseudocysts include rupture of mural vessels due to increased intracystic pressure or enzymatic erosion, as well as rupture of pseudoaneurysms in adjacent arteries weakened by inflammation^[^8,9^]^. Hemorrhage patterns vary and include bleeding confined to the cyst, and rupture into the peritoneal cavity, gastrointestinal tract, bile duct (hemobilia), or pancreatic duct (hemosuccus pancreaticus)^[^6,8^]^.

For pseudoaneurysm-related hemorrhage confined to the cyst, TAE is the preferred first-line therapy^[^3,4^]^. However, rebleeding occurs in up to 37% of cases^[^5,9^]^. Drainage after TAE is an option but may not prevent recurrence[10]. Surgical resection is indicated for failed TAE or repeated bleeding but is technically challenging because of inflammation and adhesions and carries a high risk of complications.

In the present case, recurrent hemorrhage arose from different arterial branches, with extensive SMV thrombosis likely secondary to inflammation. Initial management with TAE was chosen because of the high surgical risk and patient preference but repeated bleeding necessitated PD. The Anthron-coated catheter shunt enabled safe SMV reconstruction despite thrombosis. Although recanalization of the SMV by IVR was theoretically possible, the thrombosis was extensive, and collateral circulation was present. In addition, the coexistence of recurrent arterial bleeding made anticoagulation or IVR alone unsuitable. Therefore, definitive surgery was selected as the most appropriate strategy.

Although collateral venous pathways were observed, a non-reconstruction approach was considered potentially feasible. However, intraoperative rupture or insufficiency of these collaterals could have resulted in catastrophic intestinal congestion or hepatic ischemia. For this reason, we elected to perform portal vein reconstruction while simultaneously employing the Anthron^®^ catheter shunt. The safety and utility of this technique in preventing splanchnic congestion during clamping have been demonstrated in prior reports^[^11^]^, and in our case, it provided secure vascular control and facilitated successful reconstruction.

Regarding graft selection, we used the external iliac vein because it was the conduit with which we had prior surgical experience and reliable harvest technique. Other options, such as the left renal vein or autologous portal vein segments, have been reported^[^12^]^, but in our institution, the external iliac vein was the most feasible choice. Non-reconstruction approaches have also been described^[^13^]^; however, given the 5 cm length of thrombosis and the need to maintain portal venous flow, reconstruction was deemed necessary. Vein resections longer than 3 cm are associated with poor patency rates without grafting^[^14,15^]^, supporting our decision for interposed graft reconstruction.

To the best of our knowledge, no previous reports have described recurrent hemorrhagic pancreatic pseudocyst with SMV thrombosis managed by PD and vein graft reconstruction after multiple TAEs. This case underscores the importance of individualized treatment planning, careful follow-up after IVR, and readiness to proceed to surgery when indicated.

## Conclusion

Recurrent hemorrhagic pancreatic pseudocyst with SMV thrombosis is rare and associated with important management challenges. Although IVR can provide initial hemostasis, repeated bleeding may necessitate surgical intervention. PD with SMV reconstruction using a vein graft can be performed safely when meticulous preoperative planning and intraoperative vascular control are ensured.

## Data Availability

No datasets were generated or analyzed for this case report; therefore, data sharing is not applicable.

## SCARE guidelines

This work has been reported in accordance with the SCARE criteria (Surgical Case Report Guidelines)^[^16^]^.
